# A New Perspective for Predicting the Therapeutic Success of RFA in Solid BTNs: Quantitative Initial RFA Ratio by Contrast-Enhanced Ultrasound

**DOI:** 10.3389/fendo.2022.904459

**Published:** 2022-06-14

**Authors:** Yaqiong Zhu, Ziyu Jiao, Lianhua Zhu, Fang Xie, Qing Song, Lin Yan, Yukun Luo, Mingbo Zhang

**Affiliations:** ^1^Department of Ultrasound, The First Medical Center of Chinese People's Liberation Army (PLA) General Hospital, Beijing, China; ^2^Departments of Ultrasound, The Seventh Medical Center of Chinese People's Liberation Army (PLA) General Hospital, Beijing, China

**Keywords:** radiofrequency ablation, contrast-enhanced ultrasound, initial radiofrequency ablative ratio, volume reduction ration, therapeutic success, thyroid nodule

## Abstract

**Objective:**

The short-term therapeutic success of radiofrequency ablation (RFA) in solid benign thyroid nodules is of great concern. The aim of this study was to investigate a new method, initial radiofrequency ablative ratio (IRAR) using contrast-enhanced ultrasound (CEUS), for predicting therapeutic success of RFA in solid benign thyroid nodules (BTNs) immediately and effectively after RFA.

**Methods:**

A total of 813 nodules in 776 patients with benign thyroid nodules were treated with RFA from January 2014 to August 2018, among which 120 patients (M:F=41:79) with 120 solid BTNs (small: ≤10ml, n=57; medium: 10-30ml, n=42; large: >30ml, n=21) were enrolled in our study according to the inclusion criteria. The IRAR was defined as the ablative volume ratio immediately after RFA displayed by CEUS. The therapeutic success was evaluated at the 6-month follow-up. The relationship between the IRAR and volume reduction ratio (VRR) at 6-month was analyzed. The marginal regrowth of solid BTNs was also examined by CEUS at the 6 and 12 months of follow-up.

**Results:**

In medium and large nodules, the IRAR was significantly and positively correlated with VRR (r= 0.69, *P* < 0.001) at 6 months after RFA. There was a tendency to achieve therapeutic success (50% VRR: 55/63, 87.3%) when the IRAR exceeded 75%, and marginal regrowth was also relatively slow within 12 months after a single session treatment. No significant correlation between IRAR and VRR of small nodules was found. In conclusion, IRAR is significantly and positively correlated with VRR, which may indicate therapeutic success when it exceeds 75%.

**Conclusions:**

CEUS can be used to accurately quantify the IRAR, which is positively correlated with the VRR. Moreover, the IRAR may be used as a parameter to predict the short-term therapeutic success of RFA in solid BTNs.

## Introduction

Imaging-guided thermal ablation (IGTA) is commonly performed in clinical therapy and has been proposed for benign thyroid nodule (BTN) treatment (1–3). Among such methods, ultrasound (US)-guided radiofrequency energy thermal ablation has been used to treat solid BTNs with symptomatic or aesthetic effects; this method is highly recommended by multiple guidelines and expert consensus (4–8). Many studies, including single-centre, multicentre, retrospective and prospective studies, have demonstrated the efficacy and safety of RFA (9–12). The efficacy and safety of RFA have been evaluated at 6 months to 7 years after therapy using multiple parameters, demonstrating significant improvements in compressive symptoms, cosmetic appearance, VRR, postoperative complications, and therapeutic success (13–15).

However, for solid BTNs, the accurate quantitative ablation range immediately after the first single session of RFA treatment to predict short-term therapeutic success is of great concern since therapeutic success has been used as an indicator of RFA success during the follow-up period (7, 16, 17). At the same time, after the first session, the patient is also very concerned about whether the tumour will regrow and whether additional ablation will be needed. Approximately how long is this period after the operation? This problem may also be closely related to the initial ablation range of the nodules.

Contrast-enhanced ultrasonography (CEUS) is a widely used technique in recent years that is thought to help clarify the boundary between the ablated area and the viable area. We hypothesized that an accurate quantitative initial radiofrequency ablative ratio (IRAR) determined using CEUS could predict therapeutic success after RFA during short-term follow-up and possibly further predict the time range for additional ablation.

The purpose of this study was to determine the IRAR (CEUS quantification) threshold for predicting therapeutic success by evaluating the correlation between the IRAR and VRR and to provide a possible time range for the additional ablation of solid BTNs.

## Materials and Methods

### Patients

This retrospective study was approved by the Chinese PLA General Hospital Ethics Committee (S2019-211-01), and patient consent was waived.

**Figure 1:**
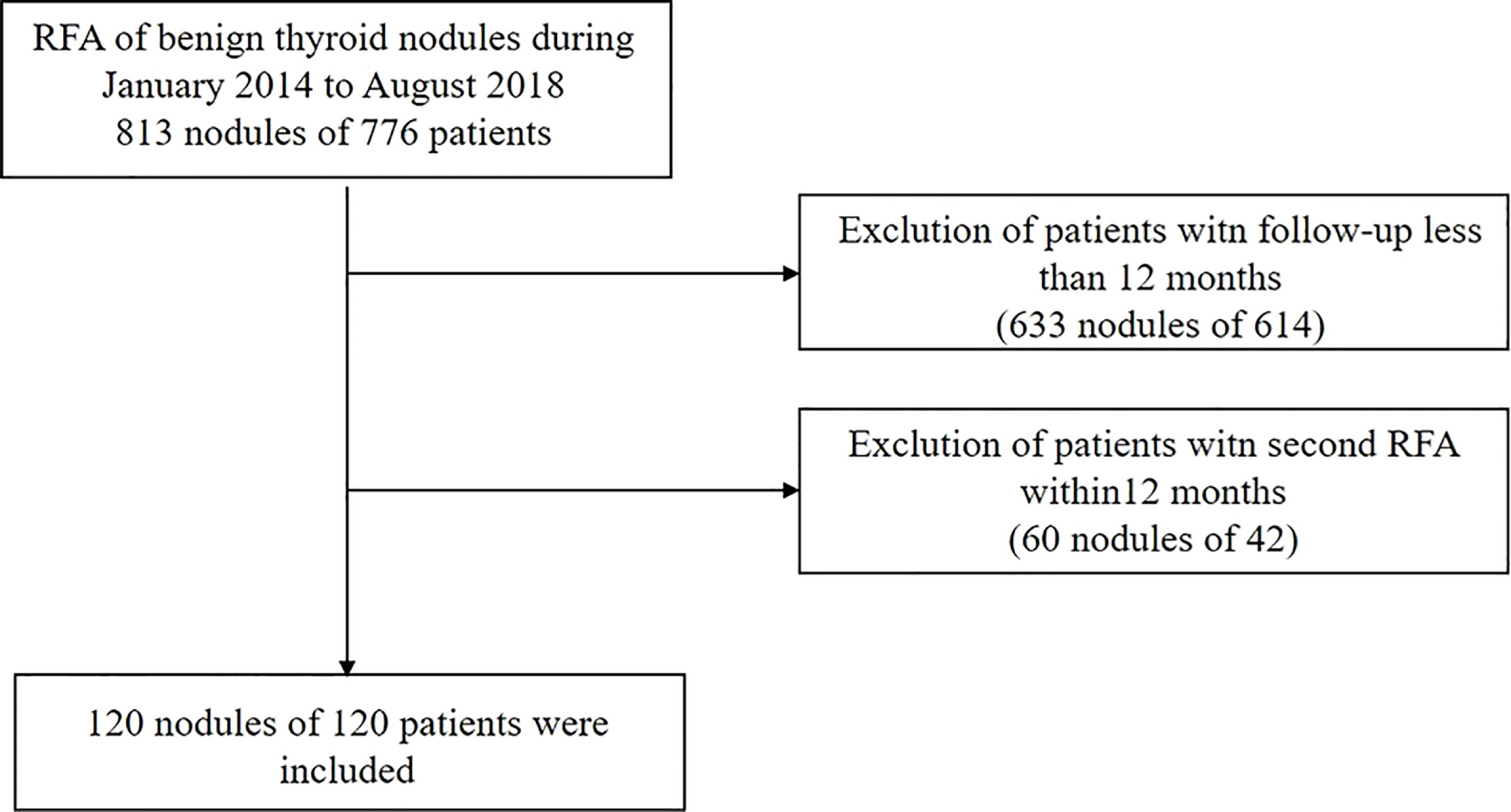
Patient Registration Flowchart. RFA, radiofrequency ablation.

A total of 813 nodules in 776 patients with BTNs were treated with RFA from January 1, 2014, to August 31,2018. According to the inclusion and exclusion criteria, 120 nodules from 120 patients were selected (**Figure 1**). All patients fulfilled the following criteria: (a) solid nodule (≤10% of fluid component); (b) two separate fine-needle aspiration (FNA) procedures confirmed a benign nodule (no more than 6 months before the procedure); (c) no malignant features on the US criteria (ill-defined margin, marked hypoechoic pattern, taller-than-wide shape); (d) single RFA treatment session; (e) no history of diffuse thyroid disease; and (f) serum thyroid hormone, thyrotropin and serum calcitonin levels within normal limits. Patients who underwent additional RFA within 12 months or with a follow-up period less than 12 months were excluded by this criterion.

### Preablation Assessment

RFA was performed on all patients after evaluation by US, FNA, and clinical and laboratory examination data. The US-guided biopsy was performed by experienced radiologists who had more than 10 years of thyroid US experience using the Siemens Acuson Sequoia 512 Ultrasound System with a 6L3 linear array probe. The largest diameter and the 2 diameters perpendicular to it (orthogonal diameters of each nodule) were measured before RFA. For each nodule, the volume was calculated using the following equation: V=πabc/6 (where V is volume, a is the largest diameter, and b and c are the 2 perpendicular diameters) (4, 18, 19). The nodules were classified into small (≤10 ml), medium (10-30 ml) and large (>30 ml) subgroups according to the baseline volume (2).

### RFA Procedure

All nodules were confirmed to be benign by analysing the results of at least two FNA biopsies. No intravenous sedatives or analgesics were used preoperatively or intraoperatively. The patients were administered local anaesthesia with 2% lidocaine at the puncture site and a thyroid anterior capsule. To avoid the chance of thermal injury, hydrodissection was necessary if the distance between the nodule and the carotid artery, internal jugular vein, recurrent laryngeal nerve, trachea or oesophagus was less than 5 mm. Under continuous US tracing, the trans-isthmic approach or direct nodule puncture approach (1) and the “moving shot” technique (4) were used when RFA was performed by an experienced radiologist (Y.K.L.) with more than 20 years of experience in thyroid interventional sonography. The RF device used was an Olympus Surgical Technologies (CelonLabPOWER, Europe, Germany, Hamburg) system with an 18-gauge bipolar radiofrequency applicator and a 15-mm active tip (CelonProSurge, micro-100-T15; Olympus Surgical Technologies, Europe, Germany, Hamburg). After RFA, each patient was observed for at least 2 hours to evaluate potential complications and side effects.

### Calculation of the IRAR

CEUS examination (Siemens Acuson Sequoia 512 Ultrasound System with a 15L8W linear array transducer) was performed immediately after RFA to observe the blood supply in the ablation area and to ensure that the ablation was as complete as possible because unablated nodule portions with vascularity have considerable potential for regrowth on follow-up. However, colour Doppler US is not sufficiently sensitive to detect small blood vessels and slow blood flow (20). To overcome these shortcomings of colour Doppler US, some authors have suggested that CEUS can be used as an auxiliary diagnostic tool for detecting underablated portions after RFA (21–24). The ablated volume was measured and calculated according to the nodule volume formula. The IRAR was defined as the ablative volume divided by the total volume of nodules immediately after RFA treatment.

### Follow-Up VRR of Nodules

The follow-up US examination was performed within 1 to 3 months postoperatively, and the second and third follow-up examinations were performed at 6 and 12 months after the single RFA treatment session. During the 12-month follow-up period, careful examination was required for early signs of nodular regrowth (or marginal regrowth), including a thickness of viable tissue within the nodules exceeding 10 mm on CEUS. Regrowth was defined as an increase in nodule volume of 50% compared to that at the previous examination (15, 25). CEUS was used to detect the ablative volume reduction and to assess early signs of nodular recurrence. Therapeutic success was defined as a VRR gets greater than 50% 6 months after RFA.

### Statistical Analysis

The results were analysed by SPSS, version 22 (SPSS, Inc., Chicago, IL, USA). Continuous variables are reported as the mean ± SD with the range. The relationship between the IRAR and VRR (at the 6-month follow-up) was analysed by Pearson correlation analysis. A *P* value less than 0.05 indicated a significant difference.

## Results

### Baseline Characteristics

The baseline characteristics of the solid BTNs and the treatment characteristics of the patients, such as the ablation duration, energy and power, are summarized in **Table 1**.

**Table 1:** Patients’ demographic data and information of nodules and operation.

Variables	Characteristics
Gender (male:female)	
Small nodules	15:42
Medium nodules	19:23
Large nodules	7:14
Age (years)	
Small nodules	49.96 ± 14.64
Medium nodules	51.74 ± 13.89
Large nodules	41.45 ± 17.36
Volume (mL)	
≤10mL (n=57)	5.67 ± 2.43
10~30mL (n=42)	17.88 ± 5.39
>30mL (n=21)	45.09 ± 14.11
RFA Duration (second)	491.4 ± 159.1
RFA energy (kJ)	3.88 ± 1.19
RFA power (W)	5.73 ± 1.37

### IRAR and Volume Changes

The mean IRAR was 82.98 ± 18.23% among all nodules, and the mean IRAR of small nodules was greater than those of medium and large nodules. Correspondingly, the VRR of small nodules at 6 and 12 months after RFA was greater than those of medium and large nodules. In brief, for the same duration of volume reduction, nodules with a smaller initial volume showed a higher VRR. The initial volume, IRAR, and VRR during the follow-up period are summarized in **Table 2**.

**Table 2:** Initial volume, IRAR, and VRR of 6 and 12 months after RFA.

	Initial volume (ml)	IRAR (%)	VRR_(6m)_ (%)	VRR_(12m)_ (%)
All nodules (120)	16.84 ± 15.73	82.98 ± 18.23	67.01 ± 12.52	79.44 ± 12.09
Small nodules (57)	5.67 ± 2.43	91.29 ± 13.22	71.32 ± 9.83	84.63 ± 10.45
Medium nodules (42)	17.88 ± 5.39	74.31 ± 22.14	66.02 ± 13.98	77.80 ± 11.50
Large nodules (21)	45.09 ± 14.11	77.97 ± 10.35	57.29 ± 10.33	68.65 ± 9.42

### Correlation Between IRAR and VRR at 6 Months After Operation

**Figure 2:**
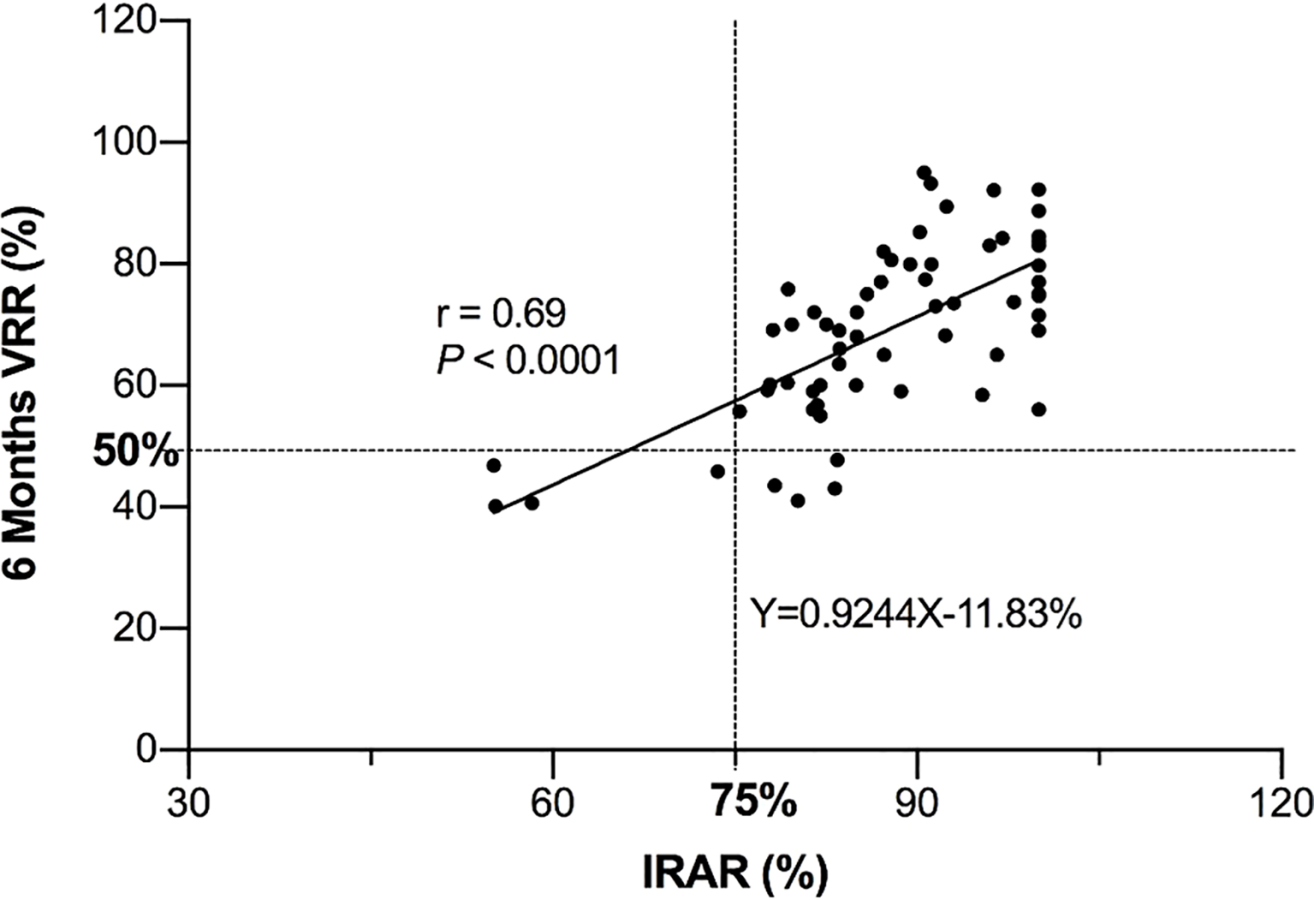
Relationship between IRAR and VRR of medium and large nodules at 6 months after RFA.

At 6 months after the operation, the correlation coefficient between the IRAR and VRR of medium and large nodules was 0.69, indicating a positive correlation (*P* < 0.001) (**Figure 2**). However, no significant correlation between the IRAR and VRR of small nodules was found, as reason may be the active tissue in small nodule tends to be completely ablated, so the IRAR of most nodules tends to reach 100%, in addition, all small nodules had VRR greater than 50% at 6 months and 12 months, which met the standard of therapeutic success.

In the figure, 4 nodules showed failure at 6 months, possibly due to the insufficient IRAR. The other 4 nodules showed failure at 6 months and adequate IRAR, which turned to therapeutic success at 12 months. The reason maybe the slow reduction of nodules.

**Figure 3:**
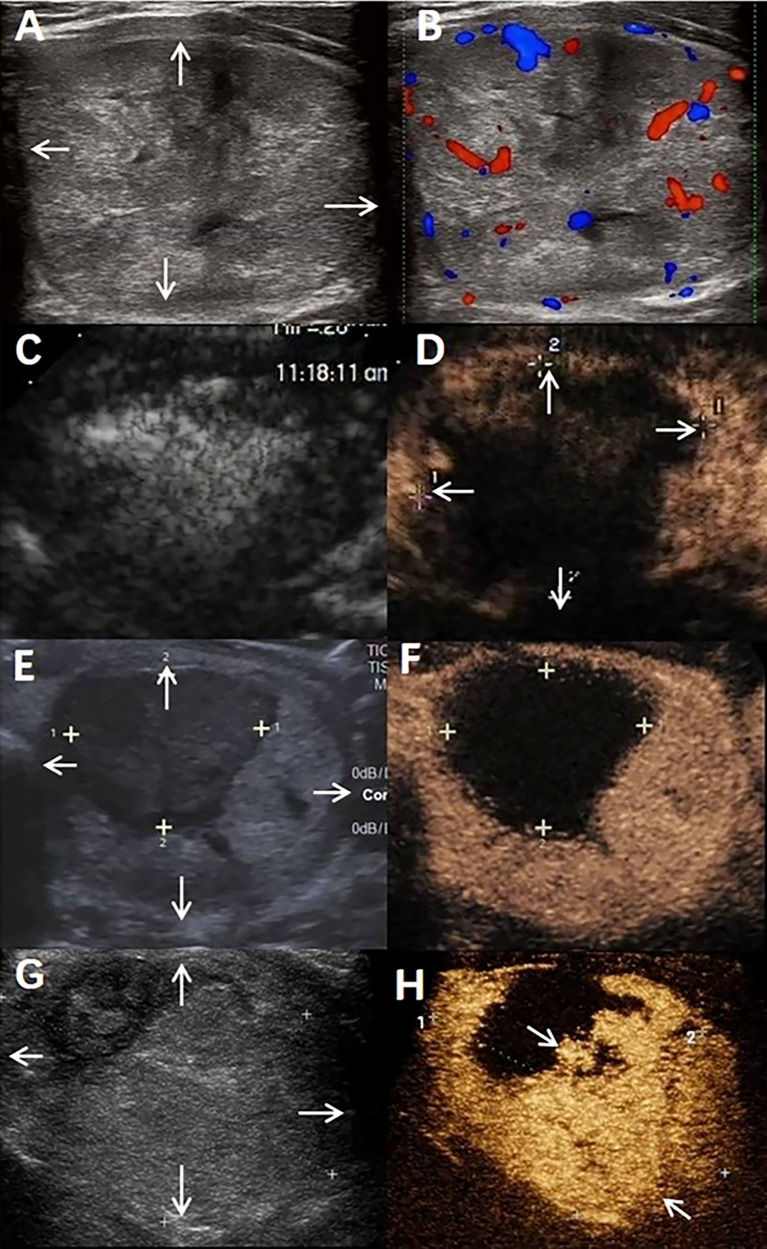
A benign thyroid nodule in a 44-year-old male treated with radiofrequency ablation (RFA). **(A)** The nodule was in the patient’s left lobe. Arrows indicate the boundaries of the nodule. The longest diameter was 6.2 cm, and the volume was 62.6 mL. **(B)** Colour ultrasound imaging of nodule. **(C)** Conventional ultrasound imaging of nodule Immediate after radiofrequency ablation. **(D)** Contrast-enhanced ultrasound imaging of nodule Immediate after radiofrequency ablation. Arrows indicate the boundaries of the treated area. The longest diameter of treated areas was 5.3cm and the volume was 36.5mL. IRAR is 58.3%. **(E)** Conventional ultrasound imaging of nodule at 6 months after radiofrequency ablation. Arrows indicate the boundaries of the nodule. The longest diameter was 5.0cm, the total volume of nodule was 37.2mL, VRR is 40.6%. **(F)** Contrast-enhanced ultrasound imaging of nodule 6 months after radiofrequency ablation. **(G)** Conventional ultrasound imaging of nodule at 12 months after radiofrequency ablation. Arrows indicate the boundaries of the nodule. The longest diameter was 5.2cm, the total volume of nodule was 35.1mL. VRR is 43.9%. **(H)** Contrast-enhanced ultrasound imaging of nodule 12 months after radiofrequency ablation. Arrows indicate the marginal regrowth of the nodule.

**Figure 4:**
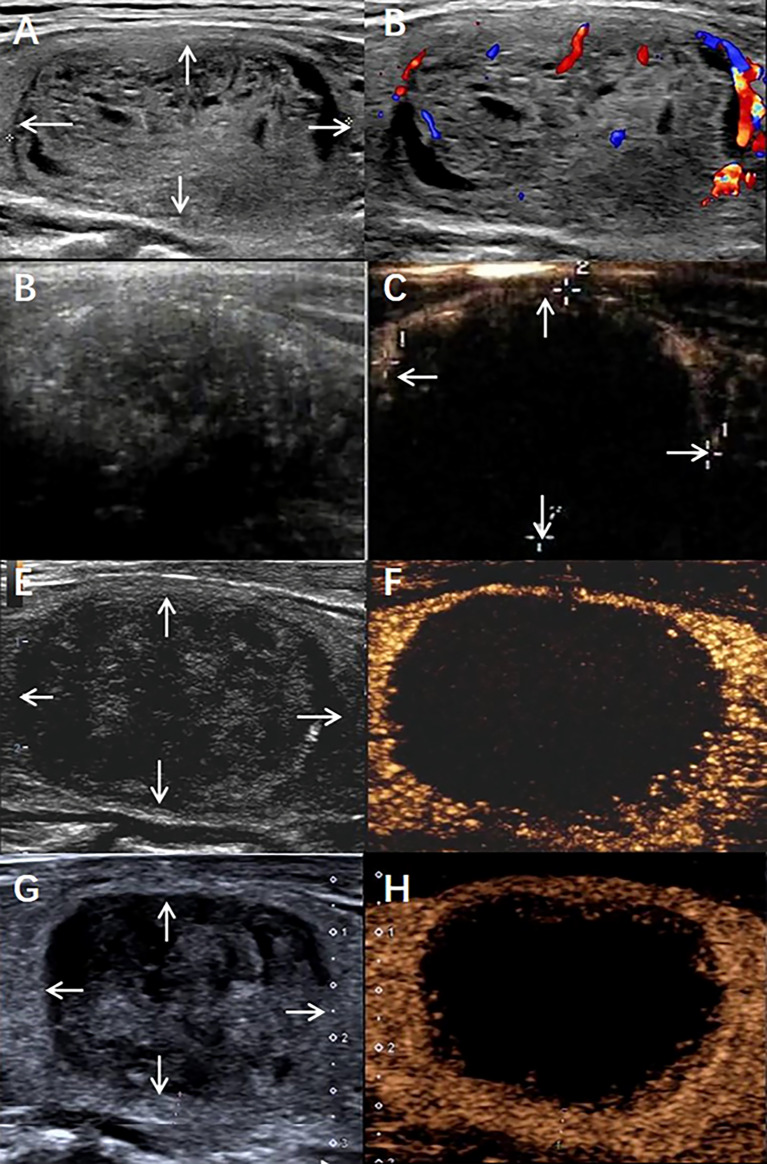
A benign thyroid nodule in a 58-year-old male treated with radiofrequency ablation (RFA). **(A)** The nodule was in the patient’s right lobe. Arrows indicate the boundaries of the nodule The longest diameter was 4.2 cm, and the volume was 21.4 mL. **(B)** Colour ultrasound imaging of nodule. **(C)** Conventional ultrasound imaging of nodule Immediate after radiofrequency ablation. **(D)** Contrast-enhanced ultrasound imaging of nodule Immediate after radiofrequency ablation. Arrows indicate the boundaries of the treated area. The longest diameter treated areas was 4.1cm and the volume was 17.7mL, IRAR is 83.7%. **(E)** Conventional ultrasound imaging of nodule 6 months after radiofrequency ablation. The longest diameter was 3.2cm, the total volume of nodule was 12.7mL, VRR is 40.6%. **(F)** Contrast-enhanced ultrasound imaging of nodule 6 months after radiofrequency ablation. **(G)** Conventional ultrasound imaging of nodule 12 months after radiofrequency ablation. The longest diameter was 2.5cm, the total volume of nodule was7.67mL., VRR is 65.2%. **(H)** Contrast-enhanced ultrasound imaging of nodule 12 months after radiofrequency ablation.

As shown in **Figure 2**, the line parallel to the x-axis represents 50% VRR, and the line parallel to the y-axis represents 75% IRAR; most of the dots are gathered in the upper right corner. Briefly, there was a tendency to achieve therapeutic success (<50% VRR at 6 months after RFA) when the IRAR exceeded 75%. **Table 3** shows eight cases of therapeutic failure, and the clinical factors of these cases are summarized. No patients with an IRAR of <75% achieved therapeutic success (cases all in the danger area), and early signs of nodular regrowth in the undertreated peripheral portion of the nodule were found at the 12-month follow-up in 4 nodules (**Figure 3**). However, there were four cases wherein the VRR was <50% at 6 months postoperatively, even though the IRAR exceeded 75%. Further follow-up revealed a further increase in the VRR at 12 months postoperatively in all 4 cases, VRR of all these nodules exceed 50%, achieve the criteria for therapeutic success, with no early signs of nodular regeneration (**Figure 4**).

**Table 3:** Nodules with <50%VRR at 6 months.

Case	IRAR (%)	6months VRR (%)	Initial volume (ml)	Reason	Marginal regrowth
1	55.1	46.9	17.9	Adjacent to vital organs	yes
2	55.3	40.1	28.4	Adjacent to vital organs	yes
3	58.3	40.6	62.6	Adjacent to vital organs	yes
4	73.6	45.9	23.8	Adjacent to vital organs	yes
5	78.2	43.5	40.7	Volume reduction slow	no (VRR:65.4%)
6	80.2	41.0	45.9	Volume reduction slow	no (VRR:67.1%)
7	83.2	43.0	49.6	Volume reduction slow	no (VRR:63.6%)
8	83.4	47.7	21.4	Volume reduction slow	no (VRR:65.2%)

IRAR, initial radiofrequency ablative ratio; VRR, volume reduction ratio.

Adjacent to vital organs: trachea, carotid artery, internal jugular vein, oesophagus, recurrent laryngeal nerve.

Volume reduction slow: High initial ablation rate and no marginal regrowth 12 months after ablation.

## Discussion

This study demonstrates that when the volume of solid BTNs was larger than 10 ml, the IRAR quantified by CEUS immediately after RFA was significantly positively correlated with the VRR at 6 month follow-up. When the IRAR of medium and large solid BTNs exceeded 75% VRR of most cases (55/63, 87.3%)would reach 50% at 6 months after operation. IRAR cannot be used as a quantitative indicator for predicting the therapeutic success of solid BTNs radiofrequency ablation. The therapeutic success is very important for the patient’s postoperative management. Lim et al. and Valcavi et al. reported the recurrence rates after RFA (5.6%) and laser ablation (9%) (13, 26). All of these recurrent cases showed the nodular marginal regrowth, which was caused by of insufficiently treatment (27). Considering the important structures adjacent to the nodules and patient tolerability, the IRAR may not reach the threshold even in the possible maximum range of ablation extent (IRAR<75% in this study). A single treatment session cannot completely ablate the periphery of the nodule; thus, nodular regrowth tends to occur since the VRR of a single treatment session. Therefore, it is known that the larger nodules are need to be treated repeatedly in order to prevent recurrence. However, despite of the many studies into this topic, there has not yet a common sense on the appropriate timing for repeated treatment of the marginal regrowth caused one undertreated session. In this study, 4 patients with an IRAR of less than 75% showed therapeutic failure (VRR<50%) at 6 months after RFA, and they were found to have marginal regrowth at the 12-month follow-up. Our results show a similar period of time after which nodular regrowth begins as that previously reported by Sim et al. (15). Such patients with an IRAR less than 75% should be informed of the necessity for close follow-up for a short period of time (at least 1 year) after RFA, and repeated ablation may be arranged based on the patients’ symptoms or their intentions if marginal regrowth occurs. In our clinical practice, the goal of RFA is usually to obtain symptom relief (9). In most cases, a single treatment session is sufficient to achieve this goal (28). However, some studies have also reported that more than one treatment sessions were performed with the aim of obtaining the greatest shrinkage (13, 29). Therefore, we need to quantify the scope of initial ablation to evaluate the adequacy of ablation, that is, to predict the success of treatment and whether RFA is needed again in the early postoperative follow-up period.

The VRR was related to the initial nodule volume (4, 5, 13). In this study, the VRRs at 12 months were 86.43 ± 10.05% in small nodules, 77.80 ± 11.50% in medium nodules, and 68.65.43 ± 9.42% in large nodules, indicating that the VRR tended to increase slowly with increasing nodule size. Moreover, for some nodules with volumes of more than 10 ml, when the IRAR was less than 75%, the VRR was low, and the phenomenon of nodular marginal regrowth was found at the 12-month follow-up. In contrast, when the IRAR was more than 75%, the VRR was relatively high, and no obvious signs of nodular regrowth were found on CEUS 12 months after RFA. Therefore, the index nodule size is an important factor in analysis of the efficacy of thermal ablation (26, 30).

The IRAR and VRR are closely related to the ablation equipment or technical factors (10). Internally cooled electrodes are used at many research centres, such as Baek, while the type of radiofrequency needles, we used may increase the heat during the thermal ablation process. However, the hydrodissection technique could minimize thermal damage to the surrounding critical structures (10). Moreover, the “moving shot” technique (31) prevents the energy source from being confined to the centre of the tumour (the target nodule be virtually divided into multiple small ablation units), allowing the energy source to constantly move to maximize the ablation area. Regarding the minimization of marginal recurrence, it is even more important to deal with the margin completely. A low IRAR indicates the presence of a large proportion of marginal viable tissue surrounding the central ablated tissue, which had been undertreated during the previous RFA session (13, 15). Although leaving a large margin is safe from the perspective of complications, leaving too much of a margin may lead to therapeutic failure, such as the VRR being lower than 50% at the 6-month follow-up. Some studies have shown that although well-treated nodules decrease in volume by 70-90% within 1–2 years (13, 32–34), incompletely treated nodules may start to enlarge 1–3 years after ablation (15, 26, 35, 36). Hence, a sufficient IRAR may ensure treatment success postoperatively and reduce the incidence of early signs of nodular regrowth (37).

The IRAR we obtained was higher than that of the results reported by Sim et al. (38), which was used to predict therapeutic success 6 months after RFA. On the one hand, it may be that our results mainly focus on medium and large nodules because no significant correlation between the IRAR of small nodules and therapeutic success was found, so the data of the small nodules were not included in the correlation analysis. On the other hand, CEUS was used to accurately quantify the ablation volume immediately after RFA, which was different from the method applied to calculate the IRAR in the study of Sim et al (38). He reported that the IRAR was estimated by subtracting the viable volume measured at the first follow-up from the total volume due to edema and haemorrhage after RFA. Therefore, the advantage of using CEUS in this study is that the ablation range and viable area can be accurately quantified.

The limitations of this study must be taken into account. First, we recognize that the sample sizes in the different subgroups were relatively small, and that additional data are required to adequately assess the correlation between the IRAR and therapeutic success in solid BTNs. Another limitation was the retrospective design of this study and its performance at a single centre. Future prospective multicentre and/or multinational studies are necessary to confirm our results.

In conclusion, CEUS can be used to accurately quantify the IRAR, which is positively and highly correlated with the VRR. If the IRAR exceeds 75%, therapeutic success may be achieved. By considering the nodule volume and the IRAR, the follow-up interval can be more systematically determined, and repeat ablation can be planned based on the patient’s symptoms or intentions if marginal regrowth occurs.

## Data Availability Statement

The raw data supporting the conclusions of this article will be made available by the authors, without undue reservation.

## Ethics Statement

This retrospective study was approved by the Chinese PLA General Hospital Ethics Committee (S2019-211-01), and patient consent was waived. Written informed consent for participation was not required for this study in accordance with the national legislation and the institutional requirements. Written informed consent was obtained from the individual(s) for the publication of any potentially identifiable images or data included in this article.

## Author Contributions

YZ and ZJ contributed equally to this work. YL and MZ contributed equally to this work. YL: conceptualization, methodology, supervision, and review and editing MZ: methodology, validation, supervision, and review and editing. YZ: methodology, formal analysis, data curation, original draft, and review and editing, and visualization. ZJ: formal analysis, data curation, original draft, and review and editing. Visualization: LZ: formal analysis, data curation, visualization. FX: formal analysis and data curation. QS: formal analysis, data curation, and original draft. All authors contributed to the article and approved the submitted version.

## Conflict of Interest

The authors declare that the research was conducted in the absence of any commercial or financial relationships that could be construed as a potential conflict of interest.

## Publisher’s Note

All claims expressed in this article are solely those of the authors and do not necessarily represent those of their affiliated organizations, or those of the publisher, the editors and the reviewers. Any product that may be evaluated in this article, or claim that may be made by its manufacturer, is not guaranteed or endorsed by the publisher.
